# Rectal Cancer Diagnosed after Cesarean Section in Which High Microsatellite Instability Indicated the Presence of Lynch Syndrome

**DOI:** 10.1155/2015/479753

**Published:** 2015-05-07

**Authors:** Tomohiro Okuda, Hiroshi Ishii, Sadao Yamashita, Sakura Ijichi, Seiki Matsuo, Hiroyuki Okimura, Jo Kitawaki

**Affiliations:** ^1^Department of Obstetrics and Gynecology, Fukuchiyama City Hospital, 231 Atsunaka-cho, Fukuchiyama, Kyoto 620-8505, Japan; ^2^ISHII Medical Clinic, Kyoto 617-0002, Japan; ^3^Department of Obstetrics and Gynecology, Graduate School of Medical Science, Kyoto Prefectural University of Medicine, Kyoto 602-8566, Japan

## Abstract

We report a case of rectal cancer with microsatellite instability (MSI) that probably resulted from Lynch syndrome and that was diagnosed after Cesarean section. The patient was a 28-year-old woman (gravid 1, para 1) without a significant medical history. At 35 gestational weeks, vaginal ultrasonography revealed a 5 cm tumor behind the uterine cervix, which was diagnosed as a uterine myoma. The tumor gradually increased in size and blocked the birth canal, resulting in the patient undergoing an emergency Cesarean section. Postoperatively, the tumor was diagnosed as rectal cancer with MSI. After concurrent chemoradiation therapy, a lower anterior resection was performed. The patient's family history revealed she met the criteria of the revised Bethesda guidelines for testing the colorectal tumor for MSI. Testing revealed that the tumor did indeed show high MSI and, combined with the family history, suggested this could be a case of Lynch syndrome. Our findings emphasize the importance of considering the possibility of Lynch syndrome in pregnant women with colorectal cancer, particularly those with a family history of this condition. We suggest that the presence of Lynch syndrome should also be considered for any young woman with endometrial, ovarian, or colorectal cancer.

## 1. Introduction

Microsatellite instability (MSI) is a hypermutable phenotype caused by the loss of DNA mismatch repair activity, and it is detected in about 15% of all colorectal cancers; 3% are associated with Lynch syndrome and the other 12% occur sporadically [1]. We report a case of colorectal cancer with MSI that may have resulted from Lynch syndrome and that was diagnosed after Cesarean section.

## 2. Case Report

**Figure 1:**
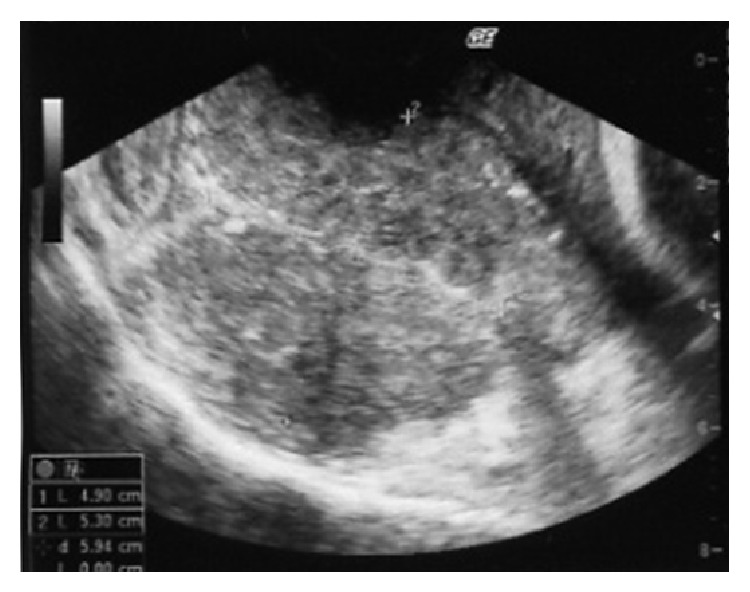
Vaginal ultrasonography demonstrated a 4.9 × 5.3 cm hypoechoic lesion behind the uterine cervix.

The patient was a 28-year-old woman (gravid 1, para 1) without a significant medical or surgical history. Her parents were alive and, according to the patient, had no significant medical history. At 35 gestational weeks, a tumor measuring approximately 5 cm was detected behind the uterine cervix on transvaginal ultrasonography (Figure 1). The mass seemed to be a uterine myoma and follow-up was planned on this basis. At 38 gestational weeks, an emergency Cesarean section was performed because the tumor had caused cephalopelvic disproportion. During the operation, the uterus and both ovaries appeared normal, but the rectum seemed swollen.

**Figure 2:**
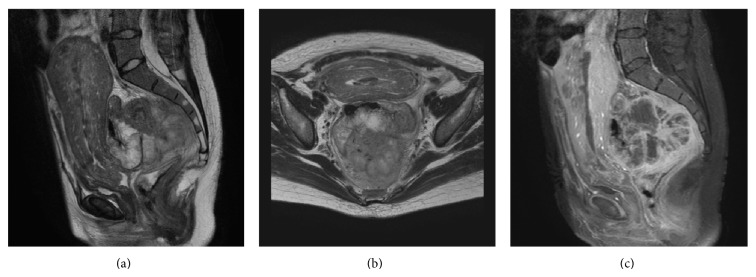
Magnetic resonance imaging (MRI) of the pelvic tumor revealed a mass measuring approximately 5 cm, behind the rectum ((a) T2 enhanced sagittal, (b) T2 enhanced axial, and (c) contrast enhanced spectral inversion recovery (CE SPIR) sagittal). The inside of this mass was stained heterogeneously with contrast medium, and the lymph nodes around the tumor were swollen. The tumor was diagnosed as rectal cancer.

**Figure 3:**
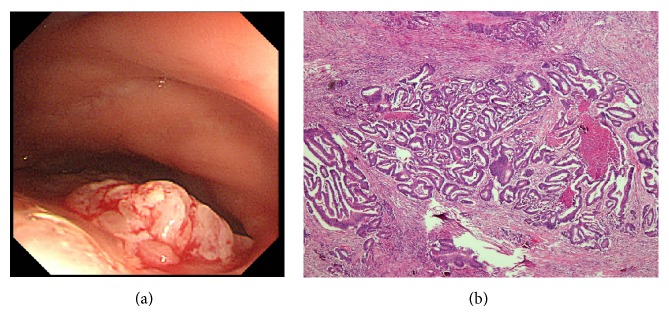
(a) Colonoscopy revealed the tumor to be an ulcerated rectal mass 20 cm from the anal area. (b) Histopathological examination of a biopsy revealed the tumor to be an adenocarcinoma of the rectum (×20).

**Figure 4:**
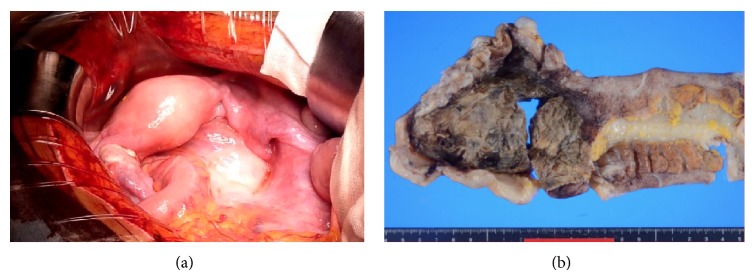
(a) Lower anterior resection with lateral pelvic lymphadenectomy was performed after chemoradiation with S-1 (TS-1, an oral fluoropyrimidine). The rectum was swollen. (b) Histopathology of the solid tumor beneath the area of ulceration prepared via formalin fixation. A localized advanced rectal cancer was diagnosed, pStage IIA (pT3 pN0 M0).

On the basis of postoperative magnetic resonance imaging (MRI), the tumor appeared to be a rectal cancer that had spread to the lymph nodes near the inferior mesenteric artery and vein (Figures 2(a), 2(b), and 2(c)). Further, colonoscopy revealed the tumor to be an ulcerated rectal mass 20 cm from the anal region, while histopathological examination of the biopsy tissue indicated the tumor to be an adenocarcinoma of the rectum (Figures 3(a) and 3(b)). No metastatic lesions were detected except for metastases to the lymph nodes on computed tomography or positron emission tomography, and no abnormalities in the upper part of the gastrointestinal tract were detected on endoscopy. The tumor markers carcinoembryonic antigen, carbohydrate antigen 19-9, and carbohydrate antigen 125 were elevated to 32.9 ng/mL, 353.6 U/mL, and 62.7 U/mL, respectively. Lower anterior resection with lateral pelvic lymphadenectomy was performed after chemoradiation with S-1 (TS-1, an oral fluoropyrimidine), and the tumor was diagnosed as pStage IIA (pT3 pN0 M0) localized advanced rectal cancer (Figures 4(a) and 4(b)).

As the patient had developed colon cancer at a young age, we reconfirmed her family medical history. Although her father was still alive and well, he had undergone surgery for colon cancer (adenocarcinoma) and a brain tumor when aged 34 and 55 years, respectively. Her grandfather and grandmother had both died of cancer (the organs affected were unclear). It became apparent that she had misunderstood the term “family history” during the earlier interview. Therefore, tests for MSI were performed during the operation. The patient fulfilled the criteria of the Bethesda guidelines for testing the colorectal tumor for MSI [2]. Using the biopsied tissue, we assessed 5 markers advocated by the National Cancer Institute for measuring MSI [3]. Of these 5 markers, BAT25, BAT26, D2S123, and D17S250 were positive, while D5S346 was negative, indicating that the tumor was MSI-high.

Although this tumor may have resulted from Lynch syndrome given the patient's family history, she did not choose to undergo germline testing for mismatch repair (MMR) gene alterations after genetic counseling.

## 3. Discussion

Colorectal carcinoma is rare in young patients [4], but even rarer in pregnancy, with a reported incidence of only 0.002% [5]. However, in these cases, the prognosis is often poor because the diagnosis is usually only made when the disease is at an advanced stage [6]. In the present case of colorectal cancer with MSI, at the time of reporting, two years has passed without recurrence in spite of advanced cancer. Elsaleh et al. have reported that tumors with MSI were more responsive to adjuvant chemotherapy than tumors without MSI [7], and Thibodeau et al. reported that patients with colorectal tumors with MSI survived longer than patients with non-MSI tumors did [8]. Similarly, Gryfe et al. reported that patients with tumors showing MSI had lower mortality rates when stratified by tumor stage, including patients with stage IV cancer [9]. Hence, the detection of MSI in a colorectal cancer is a positive prognostic factor, particularly among young patients [1]. Furthermore, MSI analysis is the first approach to identify patients with Lynch syndrome. In Japan, MSI testing has been covered by health insurance since 2006 for patients with suspected Lynch syndrome, including those who satisfy the Bethesda guidelines [10]. Lynch syndrome was a possibility in the present case based on MSI and the patient's family history. Previously referred to as hereditary non-polyposis colorectal cancer (HNPCC), this syndrome is a familial clustering of colorectal and endometrial cancers, as well as various other malignancies [11]. It is an autosomally dominant inherited genetic disease [1], and thus multiple generations can develop colorectal cancer at an early age (mean, ~45 years) [12]. Lynch syndrome is likely if a family history meets the Modified Amsterdam Criteria or revised Bethesda guidelines [2]. For patients who have a family history suggestive of Lynch syndrome, screening tests should be performed on tumor tissues to help determine the likelihood of this condition. The screening tests suggested are MSI, as described above [13], and immunohistochemical analysis. Historically, an autosomal dominant MMR deficiency leading to a tumor with MSI was assumed to be the underlying mechanism for Lynch syndrome [14]. Germline mutations in the MMR genes* MLH1*,* MLH2*,* MSH6*, and* PMS2* can lead to the development of Lynch syndrome, and heterozygosity for a mutation in one of these genes can result in increased susceptibility to cancer [12]. If a tumor shows high MSI or the protein products of the above-mentioned MMR genes are detected on immunohistochemical analysis, more specific genetic testing should be considered. Giardiello et al. published guidelines for the genetic evaluation and management of Lynch syndrome [15]. Currently, in Japan, testing companies can be commissioned to analyze the* APC*,* HLH1*,* MSH2*,* MSH6*,* PMS2*,* BRCA1*,* BRCA2*,* MEN1*,* RET*, and* HL* genes, but the costs are very high and range from hundreds to more than 3,000 USD. The costs vary depending on a number of factors including gene type and a carrier diagnosis. The test results also need to be assessed and interpreted with care, and subsequent indications need to be carefully considered. In the United States, since the enactment of the Genetic Information Nondiscrimination Act of 2008 (GINA), discrimination based on genetic information is legally prohibited in health insurance enrollment and employment. However, in Japan, currently there are no laws protecting individuals and their family from any possible misuse of genetic information. Due to this, there is ambiguity in the process of handling genetic information under medical settings. Although some pathological mutations have been characterized, it is not always clear whether other changes affecting the reading frame of these genes actually have pathological consequences [10]. The genetic diagnosis of Lynch syndrome requires a germline mutation in one of the MMR genes [16]. However, the patient in the present case did not wish to undergo germline testing for MMR genes despite genetic counseling.

If Lynch syndrome had been suspected on the basis of a careful review of this patient's family medical history, she might have been diagnosed at an earlier stage. The American Cancer Society guidelines recommend colonoscopy beginning at an earlier age for high-risk individuals [17]. Furthermore, annual colonoscopy programs performed at the age of 25 years in patients with families that have at least 3 relatives with a history of colorectal cancer or other HNPCC-related tumors have been reported to be highly effective in the early detection of colorectal cancer [18]. Additionally, a group of European experts recommended a 3-year gap between colonoscopies because this time interval has proven effective for the detection of this condition [19]. Based on these studies, the surveillance of colon cancer in Lynch syndrome patients could help to extend their survival. Currently, early cancer screening is encouraged if there has been a case of juvenile gastrointestinal cancer, endometrial cancer, or ovarian cancer with suspicion of Lynch syndrome in the family history, or breast cancer with familial aggregation. However, genetic testing is only recommended for those with a family history suggestive of Lynch syndrome, as most colorectal cancers are sporadic [20]. Even if juvenile colon cancer is found to have occurred in a close relative during a prenatal care interview, it only would indicate the need for caution, not immediate action. Typically, colonic fibroscopy is not advised during pregnancy unless there are symptoms such as gastrointestinal bleeding. The patient in the present case did not complain of melena or other symptoms that would cause us to suspect colon cancer; therefore, no special treatment was provided. In addition, the tumor expanded so rapidly at term that we thought it was uterine fibroids. Only the MRI findings lead us to suspect colon cancer, thus leading to diagnosis and treatment.

In conclusion, we report a case of rectal cancer with MSI detected in the postpartum period that was suggestive of Lynch syndrome. To the best of our knowledge, no previous study has documented colorectal cancer resulting from Lynch syndrome during pregnancy. In the experience of most physicians specializing in familial cancer, Lynch syndrome is underdiagnosed [21]. The mortality from familial cancer that develops during pregnancy including Lynch syndrome can be reduced by translating acquired genetic knowledge (e.g., family history) into clinical practice. It is important to consider the possibility of a hereditary cause, including Lynch syndrome, and hence the need for surveillance in cases of familial cancer. We also suggest that gynecologists should consider the possibility of Lynch syndrome in cases of young patients with endometrial, ovarian, or colorectal cancers.
